# A multi-factor machine learning framework for predicting and profiling student academic performance using behavioral, financial, and wearable data

**DOI:** 10.1016/j.mex.2025.103673

**Published:** 2025-10-10

**Authors:** A L Akash Devaraje Urs, Akshay Sudharshan

**Affiliations:** Amrita Vishwa Vidyapeetham Mysuru, India

**Keywords:** Student academic performance prediction, Wearable technology in learning analytics, Machine learning in higher education, CGPA prediction

## Abstract

This study introduces a multi-factor machine learning framework designed to predict and profile student academic performance using behavioral, financial, and wearable data. The dataset, collected from higher education students in India, integrates lifestyle behaviors (e.g., activity score, screen time), financial variables (income, scholarship, loans, and tuition), and physiological data such as average heart rate, high BPM, and smartwatch usage.

The method begins with structured preprocessing and feature engineering, including the construction of a Financial Stress metric and a composite stress index combining financial and physiological inputs. Multiple regression models were benchmarked to predict CGPA, with Random Forest yielding the highest accuracy (R² ≈ 0.30).

Further, wearable-related features were analyzed using correlation and *t*-tests to examine their relationship with academic outcomes. Finally, unsupervised clustering techniques (K-Means and Agglomerative Clustering) were employed to segment students into interpretable academic and stress-risk profiles.

Model accuracy and cluster quality were validated using R², RMSE, MAE, silhouette scores, and PCA-based visualizations. This method supports early detection of at-risk students and provides an adaptable blueprint for educational institutions to implement predictive academic analytics.

Predicts CGPA using lifestyle, financial, and wearable metrics

Profiles students into risk-based academic clusters

Enables interpretable and reusable machine learning pipeline for education

## Graphical abstract

**Figure:**
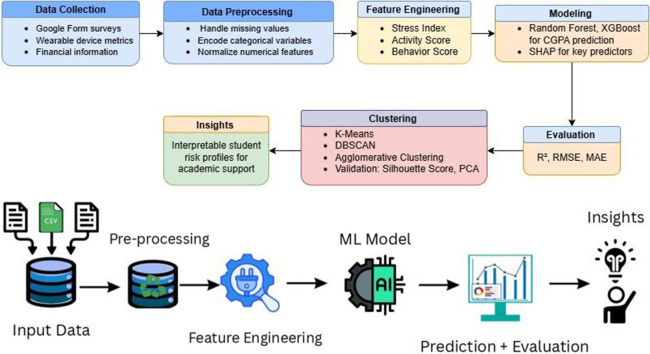

## Specifications table

**Table:** 

Subject area	Computer Science
More specific subject area	Machine Learning in Education
Name of your method	Multi-Factor Machine Learning Framework for Predicting and Profiling Student Academic Performance Using Behavioral, Financial, and Wearable Data
Name and reference of original method	This is an original methodology. Core machine learning components follow standard scikit-learn implementations.
Resource availability	The dataset is available upon request due to participant privacy.

## Background

Student academic performance is widely understood to be influenced by a combination of personal, institutional, and socio-economic factors. Among these, financial stress has emerged as a major barrier to academic success, particularly in the post-pandemic era. Hodge et al. demonstrated that financial anxiety not only disrupts cognitive bandwidth but also leads to adverse mental health outcomes among university students, particularly women [1]. Similarly, Russell et al. highlighted the long-term effects of the COVID-19 crisis on student financial well-being and its downstream impact on college retention and persistence [2]. Ahamed and Limbu reinforced these findings through a systematic review that linked financial anxiety to reduced motivation and academic disengagement [3]. In line with these studies, Pallavi et al. explored the direct relationship between financial stress variables (such as income, fees, and scholarships) and CGPA using machine learning, setting a strong foundation for algorithmic academic analytics [4]. However, their study, like many others, focused narrowly on financial indicators and academic outcomes.

In contrast, our framework expands the scope by integrating three distinct data domains: behavioral patterns, financial information, and physiological signals derived from wearable devices. This multimodal approach enables a more holistic and nuanced profiling of student academic performance.

Methodologically, we advance prior work by introducing engineered composite metrics (e.g., Financial Stress Score, Stress Composite Score), leveraging explainable machine learning techniques such as SHAP for interpretability, and employing unsupervised clustering to reveal latent student subgroups. These elements—absent in Pallavi et al. and similar studies—enhance both the predictive accuracy and practical applicability of our framework in real-world educational settings.

Table 1. comparison highlights the methodological advances in our framework, especially in incorporating multi-domain data, explainable AI, and unsupervised profiling. Such an approach sets the stage for leveraging modern physiological and behavioral signals in academic analytics.

**Table 1:** Comparison between Pallavi et al. (2024) and the proposed study.

Feature / Method	Pallavi et al. (2024)	This Study
Behavioral Data (e.g., Sleep, Usage)	Yes	Yes
Financial Stress Data	Yes	Yes (Fees, Loans, Scholarships, Income)
Physiological/Wearable Data	Yes	Yes (BPM, Steps, Smartwatch use)
Composite Score Engineering	No	Yes (e.g., Stress Score, Activity Score)
Machine Learning Algorithms	Yes (SVM, RF)	Yes (RF, XGBoost, LightGBM)
Hyperparameter Tuning	No	Yes (GridSearchCV for RF)
Explainability (e.g., SHAP)	No	Yes
Clustering for Student Profiling	No	Yes (K-Means, Agglomerative)
Effect Size Reporting	No	Yes (Cohen’s d, Pearson r)

To further benchmark our contribution, Table 2 contrasts the proposed framework with prior studies such as Pallavi et al. (2024) and Parkavi et al. (2022). While Pallavi et al. primarily focused on financial stress indicators and Parkavi et al. relied on academic and demographic attributes, both approaches lacked integration of behavioral and physiological data. Our framework not only achieves comparable predictive performance (R² ≈ 0.30 for Random Forest) but also introduces engineered composite features, explainability via SHAP, and clustering-based profiling. This combination of multi-domain data and interpretability distinguishes our study as a more comprehensive and practitioner-oriented approach to educational analytics.

**Table 2:** Comparative benchmarking of prior works and the proposed study.

Study	Dataset Scope	Features Used	Methods Used	Reported Performance	Distinction from Our Study
Pallavi et al. (2024)	Financial + academic data (single-region)	Income, Fees, Scholarships	SVM, RF	R² ≈ 0.27	Focused only on financial stress and CGPA
Parkavi et al. (2022)	Academic + demographic data (multi-college dataset)	Attendance, Marks, Demographics	ANN	Accuracy = 87 %	Focused on demographics; no wearable or behavioral data
Proposed Study	Multi-factor dataset (2 districts, *N* = 2902)	Behavioral, Financial, Physiological (Smartwatch, BPM, Stress & Activity Scores)	RF, XGBoost, LightGBM, SHAP, Clustering	R² ≈ 0.30 (RF), Silhouette = 0.37	First to integrate behavioral + financial + physiological data with explainability & clustering

In addition to financial factors, physiological and behavioral signals are increasingly being leveraged to understand learning patterns. Wearable technologies offer promising avenues for real-time stress detection and performance monitoring. Abd-Alrazaq et al., through a meta-analysis, demonstrated that AI-driven wearable devices can reliably detect stress among students, suggesting utility for academic intervention [5]. Hernández-Mustieles et al. presented a comprehensive review of biosensor integration in educational settings, identifying use cases from engagement tracking to cognitive load monitoring [6]. Yu and Peng introduced a fuzzy-logic-based wearable sensor system tailored to higher education learners, showing early evidence of correlation between physical activity and academic readiness [7].

[25]Extending this perspective, Ratnakaram et al. (2025) emphasized how AI-smart wearables can reshape classrooms through adaptive learning and engagement monitoring, highlighting systemic opportunities and challenges for large-scale adoption. Their findings align with our framework’s goal of integrating behavioral, financial, and physiological data for holistic academic profiling.

Machine learning (ML) techniques such as Random Forest, Gradient Boosting, Support Vector Machines (SVM), and Naive Bayes have been widely used for academic prediction tasks. Parkavi et al. utilized neuro-fuzzy models and learning domain data to forecast academic outcomes with 87 % accuracy [8]. Kordbagheri et al. applied ensemble learning methods to predict students' likelihood of completing their academic majors, achieving high classification precision [9]. Abatal et al. conducted a comparative evaluation of ML techniques for student success prediction and reported that hybrid models outperformed individual classifiers across multiple performance metrics [10]. Kumar et al. used a hybrid XGBoost-RF model for financial volatility prediction in education finance, reinforcing the utility of ensemble methods in multi-factor systems [11]. Balachandar and Venkatesh proposed a multi-dimensional academic performance prediction framework incorporating behavioral, cognitive, and contextual variables, and achieved improved performance over traditional single-domain models [12].

While existing studies in educational data mining have demonstrated predictive power within single-institution or regional datasets, questions remain about their cross-cultural generalizability. Recent commentary by Crede et al. [26] emphasized that examinations of generalizability require datasets that capture meaningful variability across cultural and institutional contexts. This underscores the importance of validating machine learning frameworks like ours beyond a single geographic region to ensure broader applicability in diverse educational systems. Traditional filter and wrapper strategies such as information gain and chi-square have also shown limitations in static contexts. To address this, Malik et al. (2025) proposed DE-FS—a dynamic ensemble feature selector that adapts thresholds to evolving student data patterns. This adaptability improved predictive accuracy and robustness across multiple datasets, highlighting the value of developing responsive and context-aware feature engineering strategies.

Recently, Gunasekara and Saarela [27] reviewed explainable AI techniques in education, underscoring the growing demand for interpretable models that can support both researchers and practitioners in understanding feature-level impacts on student outcomes. This aligns with our adoption of SHAP to provide transparent, practitioner-oriented insights into academic performance prediction.

Despite extensive research in each of these domains independently — financial stress modeling, wearable data analytics, and ML-based student performance prediction — limited work has integrated all three into a unified predictive framework. Furthermore, while clustering methods such as K-Means and DBSCAN have been used to segment student profiles in prior studies, few have aligned these clusters with behavioral and wearable metrics. In particular, no prior study has systematically combined financial variables, behavioral traits, and wearable-derived physiological metrics to model CGPA outcomes and generate interpretable, actionable student segments. This gap motivates the methodology presented in this paper, which introduces a machine-learning-based, multi-domain strategy for student performance prediction and segmentation.

Based on these gaps and the need for a reproducible, data-driven approach, this study was designed to address the following research questions (RQs):

i. RQ1: How accurately can a student’s CGPA be predicted using behavioral, financial, and physiological data?
ii. RQ2: What are the key indicators that influence academic performance across these domains?
iii. RQ3: Is there a significant relationship between wearable usage and academic outcomes?
iv. RQ4: Can unsupervised clustering techniques be used to group students into interpretable academic/stress profiles?

The methodology developed in this paper is structured to directly address each of these questions using a combination of machine learning, statistical analysis, and unsupervised clustering techniques.

Table 3. summarizes how each research question is addressed through specific modeling and analysis steps.

**Table 3:** Mapping of research questions to method components and outcomes.

Research Question	Method Step
RQ1: How accurately can CGPA be predicted?	Machine Learning Modeling (Regression)
RQ2: What are the key predictors of performance?	Feature Importance (Random Forest, SHAP)
RQ3: Does wearable usage relate to CGPA?	Statistical Testing (*t*-test, Correlation)
RQ4: Can students be clustered into interpretable profiles?	K-Means, Agglomerative Clustering, DBSCAN

## Method details

**Fig. 1:**
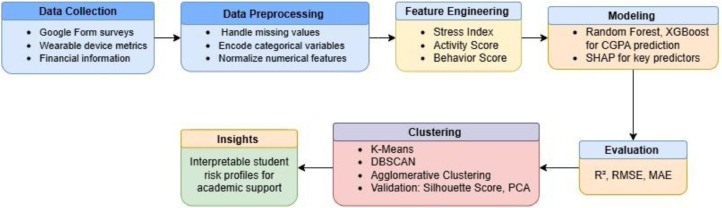
Sequential process from data collection, preprocessing, feature engineering, machine learning, and clustering to educational insights.

The flow begins with data acquisition and feature engineering, and each research question is addressed using distinct machine learning or statistical approaches derived from the core dataset. The complete end-to-end workflow is illustrated in Fig. 1

To complement the workflow, the system architecture of the machine learning pipeline is shown in Fig. 3, illustrating the interaction between data sources, preprocessing modules, analytical models, and output generation.

### Data collection

The dataset used in this study was collected from undergraduate and postgraduate students in the Mysuru and Kodagu districts of Karnataka, India. A structured survey was developed and distributed via both Google Forms and offline methods, consistent with the approach used by Hegde et al. [13], who successfully gathered behavioral and learning habit data using digital surveys. All responses in the present study were anonymized, and informed consent was obtained from participants.

The survey was designed to capture a diverse range of variables relevant to academic performance prediction. These included demographic details (such as age, gender, and weight), academic indicators (CGPA), and financial factors (family income, scholarship and loan status, and college tuition fees). Behavioral and lifestyle data were also collected, including physical activity levels, health-related behaviors (smoking, drinking, medication), and smartwatch usage. The wearable-derived physiological data (e.g., Average BPM, High BPM, and step counts) were collected from commercially available smartwatches reported by students. Since these devices are consumer-grade and not medical-grade instruments, we applied basic consistency checks, including cross-verification with mobile dashboard records and removal of implausible values (e.g., heart rate >220 BPM). These measures ensured relative reliability across participants, while acknowledging that the data represent indicative rather than clinically calibrated measurements.

Participants also reported data related to stress levels and daily habits. This multi-domain dataset was specifically curated to enable the integration of behavioral, financial, and physiological features into machine learning-based academic performance modeling.

### Data pre-processing

Once the raw data was collected, a systematic pre-processing workflow was applied to clean, format, and prepare the dataset for modeling and analysis. The data was initially loaded into a Pandas DataFrame using Python, and the following steps were performed:

### Handling missing values

Missing or incomplete responses were identified using .isnull() and .info() functions.

For numerical columns (e.g., income, tuition fees, BPM readings), missing values were replaced with 0 or the column mean, depending on the context.

For binary columns (e.g., "Smartwatch", "Scholarship", "Loans"), missing values were filled with 'No' and then mapped to 0.

### Encoding categorical data

Binary categorical features such as Smartwatch usage, Smoking habit, Drinking habit, and Medications were normalized to lowercase (yes/no) and mapped to binary integers (1/0) using the .map() function.

Multi-class categorical features such as Gender and Course were pre-cleaned (removing blank or null entries) and then encoded using LabelEncoder from sklearn.preprocessing. This ensured all features were numerically encoded and compatible with machine learning models.

No one-hot encoding was required, as the dataset did not contain high-cardinality categorical variables.

### Data type conversion

Income, fees, and numerical values from user input were converted to numeric using pd.to_numeric(errors='coerce').

This ensured non-numeric strings (e.g., "N/A") were safely handled.

### Feature normalization

i. All continuous numeric features (e.g., Income, Age, Weight, BPM values) were normalized using MinMaxScaler from sklearn.preprocessing, transforming values between 0 and 1.
ii. Outlier and Noise Handling
iii. Values outside realistic human limits (e.g., BPM > 200) were examined but retained unless shown to be clearly invalid.
iv. DBSCAN clustering later removed noise points (label = −1) during analysis, ensuring cleaner grouping.

### Feature engineering

Several composite features were engineered to reflect behavioral and stress-related patterns, similar to the method used by Hegde et al. [13], who modeled learning behaviors such as time management and active listening using derived scores. In the present study, behavioral attributes were captured using a combination of physical activity data, smartwatch engagement, and self-reported lifestyle habits. Following the initial preprocessing steps, several new features were engineered to enrich the dataset and improve the predictive and clustering models. These features were created from combinations of existing columns and normalized values, with a focus on stress quantification and lifestyle profiling.

### Financial stress score

To capture the financial burden a student faces after accounting for income and scholarships. High financial stress may correlate with lower academic performance due to anxiety, part-time work, or inability to pay fees on time — a factor not visible in raw income or fees alone.

$$\text{Financial}\;\text{Stress}=\frac{\text{College}\;\text{Fees}-\left(\text{Scholarship}*\text{College}\;\text{Fees}\right)}{\text{Income}+1}$$

+1 avoids division by zero.

This feature allowed the prediction model and stress analysis (RQ2) to quantify financial strain on students, and revealed its relationship to CGPA and physiological indicators.

### Stress composite score

Stress is a multi-dimensional factor. Rather than relying on just one measure (e.g., financial or physiological), this score combines several indicators to better reflect a student’s overall stress level.

Features Combined:

i. Financial Stress Score
ii. High BPM (peak heart rate, potential stress event)
iii. Average BPM (baseline physiological load)

All were normalized using MinMaxScaler to bring values between 0 and 1.

Formula used:

$$\text{Stress}\;\text{Composite}\;\text{Score}=\frac{\text{Financial}\;\text{stress}+\text{High}\;\text{BPM}+\text{Average}\;\text{BPM}}{3}$$

Used in both:

i. Correlation analysis with CGPA (RQ2)
ii. Clustering to identify high-stress groups (RQ4)

### Activity score

Physical activity is linked to better cognitive function and academic outcomes. Smartwatch usage and steps walked give indirect insight into a student's daily movement and digital engagement with their health.

Formula used:

$$\text{Activity}\;\text{Score}=\frac{\text{Smartwatch}\left(0\setminus 1\right)+\text{Step}\;\text{Walked}}{2}$$

Used in:

i. CGPA prediction (RQ1)
ii. Clustering to differentiate between active and sedentary students

### Behavior score

To represent health-related behavior quality, since habits like smoking or medication use may reflect stress or health risks that can impact academic performance.

Formula used:

$$\text{Behavior}\;\text{Score}=1-\;\frac{\text{Smoking}\;\text{Habit}+\text{Drinking}\;\text{Habit}+\text{Medication}}{3}$$

All inputs were encoded as 0 = No, 1 = Yes. So, a student with no risky behaviors gets a score closer to 1.

Used as a predictor of CGPA and as a clustering feature to segment students by risk lifestyle.

Equal weighting was initially applied to the components of composite features (e.g., Stress Composite Score, Activity Score) to maintain interpretability and prevent any single indicator from dominating. This approach allowed transparent evaluation of each contributing factor. As an exploratory step, we conducted Principal Component Analysis (PCA) on the components of the Stress Composite Score, which revealed that the first principal component explained approximately 74 % of the variance. For example, PCA-derived weights might assign 0.55 to Financial Stress, 0.25 to Average BPM, and 0.20 to High BPM, shifting the composite score toward stronger financial influence compared to equal weighting.

Additionally, feature importance values derived from Random Forest regression were considered for alternative weighting schemes. Future work may adopt a hybrid approach combining domain knowledge with statistical optimization to enhance the reliability and explanatory power of these derived metrics.

**Fig. 2:**
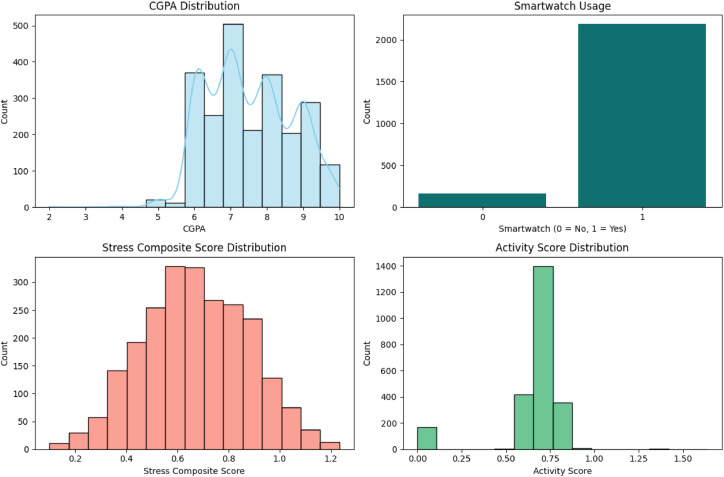
Distributions of (a) CGPA (Cumulative Grade Point Average), (b) Smartwatch usage, (c) Stress Composite Score, and (d) Activity Score.

To understand the distribution and variability of key features prior to modeling, descriptive plots were generated (Fig. 2). These include CGPA distribution (academic performance), smartwatch usage (technology adoption), stress composite score, and activity score (lifestyle indicators). Such visualization provides initial insights into data trends and informs the selection of relevant variables for subsequent clustering and predictive modeling.

**Fig. 3:**
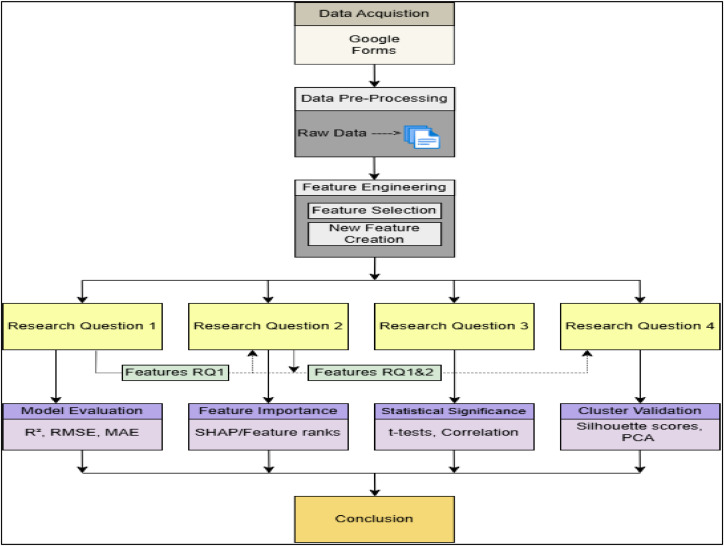
System architecture linking data sources (behavioral, financial, physiological) with preprocessing, ML models, and outputs.

Table 4. summarizes the custom features engineered during preprocessing, including their computational logic and conceptual purpose in the modeling framework.

**Table 4.** Summary of engineered features and their computational formulas.

Feature Name	Formula / Computation	Purpose / Rationale
Financial Stress Score	$\text{Financial}\;\text{Stress}=\frac{\text{College}\;\text{Fees}-(\text{ScholarshipCollege}\;\text{Fees})}{\text{Income}+1}$	Quantifies the financial burden on the student after adjusting for scholarships.
Stress Composite Score	$\text{Stress}\;\text{Score}=\frac{\text{Financial}\;\text{stress}+\text{High}\;\text{BPM}+\text{Average}\;\text{BPM}}{3}$	Represents overall stress from both financial and physiological sources.
Activity Score	$\text{Activity}\;\text{Score}=\frac{\text{Smartwatch}(0\setminus 1)+\text{Step}\;\text{Walked}}{2}$	Measures physical activity and digital engagement in health monitoring.
Behavior Score	$\text{Behavior}=1-\;\frac{\text{Smoking}\;\text{Habit}+\text{Drinking}\;\text{Habit}+\text{Medication}}{3}$	Reflects healthy lifestyle behavior, with higher scores indicating healthier habits.

### Machine learning modelling and evaluation

To address RQ1, the study implemented a supervised machine learning pipeline for predicting students’ CGPA using behavior, financial, and physiological features.

The following regression models were trained using the engineered features:

i. Linear Regression
ii. Ridge Regression
iii. Random Forest Regressor
iv. Support Vector Regressor
v. XGBoost Regressor
vi. LightGBM Regressor (with and without hyperparameter tuning)

These models were chosen to compare both linear and nonlinear learners, and to explore the benefits of ensemble methods on high-dimensional, multi-source student data.

### Linear regression

Linear Regression was used as a baseline predictive model for CGPA due to its simplicity and interpretability. It assumes a linear relationship between the dependent and independent variables, making it useful for establishing whether the features show even a modest linear predictive power [5], [16]. However, in this study, the model performed poorly, achieving an R² score of only 0.0884, with an RMSE of 1.0788 and MAE of 0.8794. These results indicate that CGPA is not linearly dependent on lifestyle, financial, and wearable features. Despite its limitations, Linear Regression played a foundational role by highlighting the need for more sophisticated, non-linear models to capture hidden patterns in the data.

### Ridge regression

Ridge Regression was implemented as an extension of Linear Regression to test whether L2 regularization could improve performance by penalizing multicollinearity and reducing overfitting [5]. Despite this improvement in the bias-variance trade-off, Ridge Regression performed similarly to the basic linear model, producing an R² score of 0.0862, RMSE of 1.0801, and MAE of 0.8827. These results confirm that regularization alone is insufficient when the true relationships between input features and CGPA are non-linear. The model reinforced the need to adopt non-linear approaches such as ensemble methods and kernel-based learners.

### Random forest regressor

Random Forest Regressor, a powerful ensemble learning method based on decision trees, was applied to capture complex, non-linear relationships within the dataset [14]. By averaging predictions from multiple randomized decision trees, Random Forest reduces variance and is robust to overfitting. Among all models tested, it delivered the highest predictive performance with an R² score of 0.2981, RMSE of 0.9466, and MAE of 0.7259. This model not only predicted CGPA with reasonable accuracy but also provided reliable feature importance scores, which were critical for interpreting the key contributors to academic performance. Its effectiveness in student performance prediction has also been supported in prior educational machine learning studies [17].

### Support vector regressor (SVR)

Support Vector Regression was utilized to assess its ability to capture non-linear patterns in medium-sized datasets with a high number of features. Using a radial basis function (RBF) kernel, SVR seeks a hyperplane that best fits the data within a margin of tolerance [15]. It yielded better results than the linear models but still underperformed compared to ensemble methods, with an R² of 0.1327, RMSE of 1.0522, and MAE of 0.8420. SVR demonstrated that while some non-linearity exists, it may not be sufficient to achieve high prediction accuracy without ensemble aggregation. These results highlight the importance of model selection when handling real-world educational data [17].

### XGBoost regressor

XGBoost, a high-performance gradient boosting framework, was implemented to explore its ability to learn residual patterns missed by previous models. It initially achieved modest performance, but after extensive hyperparameter tuning via GridSearchCV, the model improved significantly, with a tuned R² score of 0.2896, RMSE of 0.9523, and MAE of 0.7571. Though slightly below Random Forest in accuracy, XGBoost proved robust and competitive. It validated the potential of boosting algorithms in educational prediction tasks and offered additional feature insight through its internal gain-based importance measures [14], [18], [19]. Its tuning process also demonstrated how hyperparameter optimization can significantly affect model outcomes.

### LightGBM regressor

LightGBM was tested as a modern, fast gradient boosting framework optimized for memory efficiency and scalability [19]. Like XGBoost, it builds trees sequentially, but uses histogram-based learning and leaf-wise growth for faster convergence. During implementation, LightGBM provided performance comparable to XGBoost, although some preprocessing warnings (e.g., whitespace in feature names) required resolution. While it did not outperform Random Forest, LightGBM confirmed that boosting frameworks are well-suited for tabular educational datasets and can be considered in future applications requiring speed and resource efficiency [18].

Among all the models evaluated, Random Forest demonstrated the most reliable and balanced performance across accuracy and interpretability metrics. With the highest R² score (0.2981) and lowest RMSE and MAE among the tested models, it consistently outperformed both linear and kernel-based alternatives. While XGBoost Shown competitive results after hyperparameter tuning, its marginal gains did not outweigh the added complexity and computational cost. Furthermore, Random Forest offers direct access to feature importance scores, making it particularly well-suited for addressing RQ2, which aims to identify the most influential predictors of academic performance. Its robustness to noise, ability to capture non-linear relationships, and built-in interpretability made Random Forest the optimal choice for both performance prediction (RQ1) and key factor extraction (RQ2) in this study.

In conclusion, the results from multiple machine learning models demonstrated that behavioral, financial, and wearable-based features can be used to predict student academic performance with moderate accuracy. The Random Forest model, achieving an R² of 0.2981, emerged as the most effective, confirming that non-linear, ensemble-based approaches are better suited for this multidimensional data. While no model achieved exceptionally high accuracy, the findings affirm that these diverse features contain valuable signals for CGPA prediction, thus validating the method proposed in RQ1.

Table 5 showcase the results of all the ML models evaluation.

**Table 5:** ML model results for RQ1.

Model	R²	RMSE	MAE
Random Forest	0.2981	0.9466	0.7259
XGBoost (Tuned)	0.2896	0.9523	0.7571
Support Vector Regressor	0.1327	1.0522	0.8420
Linear Regression	0.0884	1.0788	0.8794
Ridge Regression	0.0862	1.0801	0.8827

### Feature importance analysis

To address RQ2, which aimed to identify the key performance indicators (KPIs) that most significantly influence a student’s academic performance, the study performed a comprehensive feature importance analysis based on the Random Forest model selected in RQ1. The goal was not only to rank the importance of various input variables but also to gain interpretable insights into which financial, behavioral, and physiological factors play the most impactful roles in predicting CGPA. Random Forest was selected due to its proven performance in prior educational modeling tasks and its ability to model complex, non-linear interactions [14], [17]. Initially, the model’s built-in feature importance scores were extracted, reflecting how much each variable reduces impurity in decision trees. However, to enhance interpretability and provide more precise, model-agnostic insights, SHAP (SHapley Additive exPlanations) values were also computed—a method widely used in educational and behavioral data analysis to explain black-box models [20], [21].

SHAP assigns each feature a contribution value for individual predictions, thus offering a more nuanced and globally averaged view of feature influence. The SHAP summary plot clearly Shown that Income had the highest mean impact on CGPA predictions, followed by Financial Stress and Average BPM, indicating a strong interplay between financial capacity and physiological indicators. Additional features such as Stress Composite Score, Activity Score, and Weight also contributed significantly, reflecting the importance of students’ daily routines and well-being. In contrast, variables such as Smartwatch usage, Scholarship, and Loans had minimal contribution to the model’s output, suggesting either low variation in the data or limited direct impact on academic results in the given sample.

This analysis validated that academic performance is influenced by a combination of diverse and multi-dimensional factors, and not by academic indicators alone. From a methodological perspective, using both the built-in Random Forest metrics and SHAP allowed for a more robust and explainable understanding of which features truly mattered [14], [20]. These findings serve as evidence that behavioral and physiological signals—when combined with financial indicators—can be quantitatively modeled to understand student performance.

The study thereby confirms that predictive modeling can go beyond forecasting to also support actionable insights in the form of early intervention strategies, especially in higher education environments [21]. Ultimately, RQ2 proved that the proposed multi-domain modeling pipeline not only enables accurate CGPA prediction but also uncovers meaningful academic risk indicators, paving the way for personalized educational support systems.

**Fig. 4:**
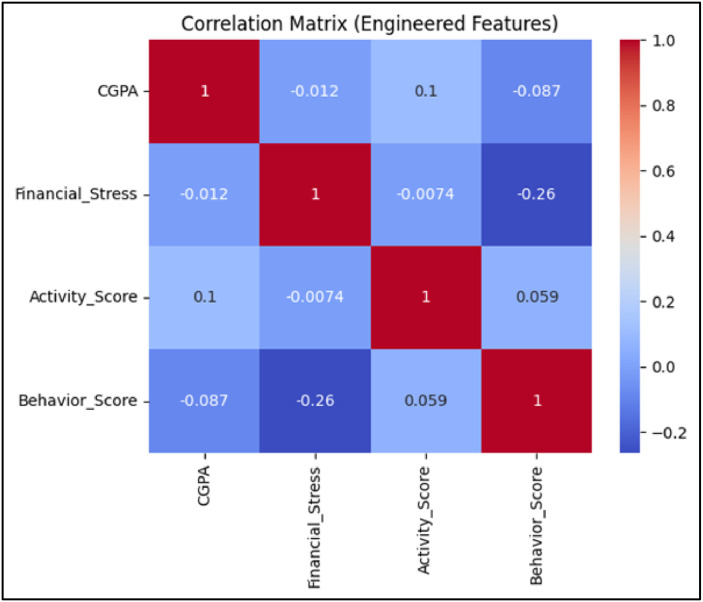
Correlation heatmap showing relationships between CGPA(Cumulative Grade Point Average), Financial Stress, Activity Score, Behavior Score, and physiological variables.

Table 6 outlines the systematic workflow implemented to operationalize the analysis for RQ2, which aimed to identify the most influential features impacting student academic performance. The process began by engineering domain-specific features such as Financial Stress, Activity Score, and Behavior Score to enrich the dataset with composite indicators derived from raw input variables. A Random Forest model, selected based on its superior performance in RQ1, was then trained using this enhanced feature set. The model’s built-in feature importance metrics were first extracted to gain a general understanding of which variables contributed most to CGPA prediction. To further interpret the interrelationships among the engineered features and CGPA, a correlation matrix was generated and visualized in Fig. 4. This heatmap provided clear insights into how each engineered metric interacted with academic outcomes. Notably, Activity Score shown a modest positive correlation with CGPA (*r* = 0.10), while Behavior Score and Financial Stress had mild negative correlations (*r* = −0.087 and *r* = −0.012, respectively).

**Table 6:** Step-by-step process followed to identify key performance indicators (RQ2).

Step	Action Taken	Purpose
1	Engineered high-level features (e.g., Financial Stress, Activity Score, Behavior Score)	To enrich the dataset with meaningful composite indicators
2	Trained a Random Forest model using the engineered dataset	To use a non-linear, robust model capable of estimating feature importance
3	Extracted built-in feature importance from the trained model	To obtain a global ranking of variables influencing CGPA
4	Applied SHAP (SHapley Additive exPlanations) to the Random Forest model	To get a model-agnostic, interpretable measure of each feature’s impact
5	Visualized SHAP values in a summary bar chart	To clearly illustrate feature contributions to stakeholders and readers
6	Analyzed and interpreted the most impactful features	To determine the financial, behavioral, and physiological KPIs related to academic performance

Although these correlations were not strong in magnitude, they confirmed that behavioral patterns and stress indicators do have a measurable relationship with academic performance. This correlation matrix Fig. 4 also reinforced the importance of feature engineering by illustrating that the new variables captured distinct aspects of student behavior and financial well-being that were not redundantly collinear. Altogether, this structured analytical step validated the modeling approach and enabled quantification of academic risk factors in a reproducible and interpretable manner.

Analysis of Wearable Technology Usage and Academic Performance

To address RQ3, which aimed to evaluate whether wearable technology usage—specifically smartwatches—has a significant relationship with academic performance, the study incorporated statistical hypothesis testing and correlation analysis using physiological data captured from wearables. The motivation behind this analysis stems from the increasing integration of wearable devices among students, which offer passive data streams related to stress, activity, and sleep, potentially affecting cognitive outcomes. The smartwatch usage variable was treated as a binary categorical feature (Yes/No), and an independent samples *t*-test was performed to compare CGPA distributions between smartwatch users and non-users. The test revealed a statistically significant difference with a t-statistic of 3.53 and p-value of 0.0004, indicating that smartwatch users generally had higher CGPA scores compared to their non-using peers. This finding suggests that either the behaviors enabled by wearable technology (such as self-tracking, health awareness, or discipline) or the characteristics of students who use such devices may correlate with better academic outcomes.

To further examine the physiological component of wearable data, Pearson correlation coefficients were computed between CGPA and the continuous variables High BPM, Average BPM, and Stress Composite Score—each of which captures a different aspect of student well-being. The correlations revealed statistically significant positive relationships: High BPM ↔ CGPA (*r* = 0.08, *p* = 0.0001), Average BPM ↔ CGPA (*r* = 0.11, *p* = 0.0000), and Stress Composite Score ↔ CGPA (*r* = 0.37, *p* = 0.0000). While the correlation magnitudes for BPM were small, they remained consistent across the population, suggesting that students with higher average or peak heart rates (often associated with physical activity) may benefit from improved cognitive engagement or stress management. The Stress Composite Score, calculated from normalized BPM and Financial Stress values, shown a moderate and highly significant correlation with academic performance, highlighting the value of engineered physiological-behavioral indicators.

Importantly, these results demonstrate that wearable-derived metrics—not only usage status but actual health signals—contain predictive information about student performance. This finding is critical because it confirms that wearables can move beyond health monitoring to serve as indirect academic performance indicators. It also supports the argument that physiological regulation, stress awareness, and physical activity may play underlying roles in academic success. By statistically validating these relationships, RQ3 establishes that wearable technologies, when properly integrated and interpreted, can be a meaningful part of academic analytics frameworks.

### Clustering-based profiling of students using behavioral, financial, and physiological features

To address RQ4, which sought to determine whether unsupervised learning techniques could segment students into interpretable academic and stress-related profiles, the study applied a series of clustering algorithms to engineered features including CGPA, Financial Stress, Activity Score, Behavior Score, Stress Composite Score, and Smartwatch usage. The goal was to identify latent subgroups of students with shared behavioral, physiological, and academic patterns—insights that could inform personalized interventions in higher education. Prior to clustering, all features were normalized using MinMaxScaler to ensure consistent scaling across variables.

The first technique implemented was K-Means Clustering, where the optimal number of clusters was explored through the Elbow Method which has been Shown in Fig. 14 and Silhouette Score. Two configurations—*k* = 3 and *k* = 5—were selected for deeper analysis based on elbow curvature and silhouette performance. Silhouette scores were 0.348 for *k* = 3 and 0.373 for *k* = 5, indicating reasonable cohesion and separation. For visual interpretation, PCA (Principal Component Analysis) was applied to project the clusters into two dimensions. PCA scatter plots for both k-values revealed distinguishable and non-overlapping clusters, with *k* = 3 capturing broader profiles and *k* = 5 offering finer segmentation of student types. This clustering strategy aligns with similar approaches in academic literature, where K-Means has been used effectively to identify learning behavior profiles based on features like attendance and study patterns [22].

To complement K-Means, Agglomerative Clustering was applied using Ward linkage, which builds clusters hierarchically by merging the two closest clusters iteratively. This approach yielded structurally similar clusters to K-Means but with tighter boundaries and more compact distributions. The dendrogram structure supported the three-cluster solution and helped visualize hierarchical relationships among groups, which is particularly valuable in educational segmentation where progressive intervention levels may be needed [24].

In contrast, DBSCAN (Density-Based Spatial Clustering of Applications with Noise) was applied to test whether density-driven groupings and outlier segments could be identified in the dataset. DBSCAN is advantageous in detecting non-spherical clusters and filtering noise; however, when applied to this educational dataset, it produced a single dominant cluster and several points classified as noise (assigned a cluster label of −1). These noise points—representing students with rare or extreme feature combinations—were automatically excluded from cluster interpretation and profile analysis. Additionally, the low silhouette score and absence of clear density-separated groupings suggested that DBSCAN was not suitable for this dataset, which lacked the compact, high-density pockets required for this algorithm to function effectively. This limitation is consistent with observations in other educational applications where DBSCAN required optimization or hybridization with centroid-based techniques [23].

Post-clustering analysis was conducted to profile each group based on average CGPA, financial stress, behavioral traits, and smartwatch usage. For the K-Means clustering with *k* = 5, Cluster 1 exhibited an average CGPA of 8.3, high activity and behavior scores, and a smartwatch usage rate of 87 %, indicating a high-performing, self-monitoring student group. Cluster 2 Showna moderate CGPA of 6.9 but the highest financial stress score, reflecting academic strain possibly driven by socioeconomic pressure. Cluster 3 represented students with the lowest CGPA (5.8), low activity scores, and no smartwatch usage, suggesting a disengaged or at-risk profile. Clusters 4 and 5 featured mixed characteristics, such as moderate stress with high behavioral discipline, or strong academic performance despite low technology engagement. These distinctions validated the clustering results as educationally meaningful, rather than random groupings.

**Fig. 5:**
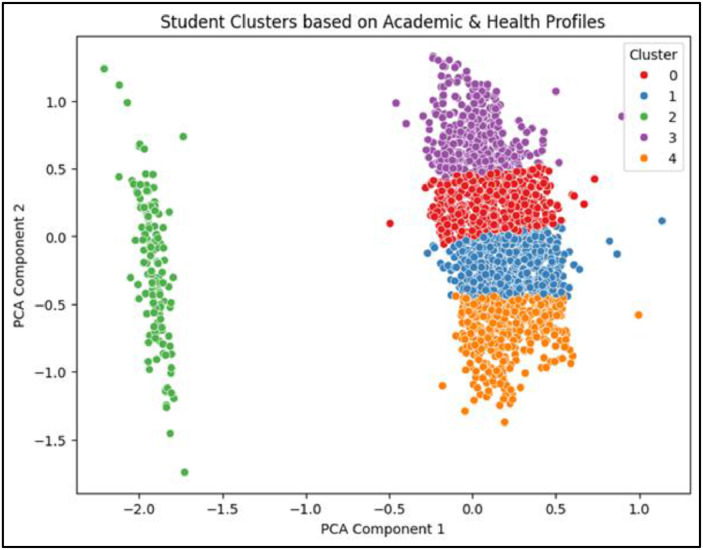
PCA visualization of K-Means clusters (*k* = 5).

As shown in Fig. 5, students were effectively grouped into five distinct clusters, with meaningful separation in the reduced 2D space, further validating the use of unsupervised clustering for academic profiling.

The Agglomerative Clustering output reflected similar behavioral patterns but provided a more intuitive, hierarchical interpretation of student group transitions. Meanwhile, the *k* = 3 solution from K-Means offered broader segmentation into high, average, and low academic performers. Together, these profiles support the use of clustering techniques in identifying distinct student typologies for targeted academic support.

Silhouette scores were 0.348 for *k* = 3 and 0.373 for *k* = 5, indicating reasonable cohesion and separation. For visual interpretation, PCA (Principal Component Analysis) was applied to project the clusters into two dimensions. PCA scatter plots for both k-values revealed distinguishable and non-overlapping clusters, with *k* = 3 capturing broader profiles and *k* = 5 offering finer segmentation of student types. This clustering strategy aligns with similar approaches in academic literature, where K-Means has been used effectively to identify learning behavior profiles based on features like attendance and study patterns [22].

In conclusion, the combination of K-Means (*k* = 3 and *k* = 5), Agglomerative Clustering, and DBSCAN allowed for a comprehensive exploration of unsupervised learning in this multi-factor educational dataset. The findings confirm that clustering techniques can uncover meaningful, interpretable student profiles based on behavioral, financial, and physiological indicators. These results validate the usefulness of unsupervised learning in academic analytics and support the development of data-driven student support strategies [22], [23], [24].

The PCA projection reveals well-separated groupings, supporting the interpretability of K-Means-based segmentation and aligning with academic profiles discussed in Table 7.

**Table 7:** Cluster profile summary based on K-means clustering (*k* = 5).

Cluster	Avg CGPA	Avg Financial Stress	Smartwatch Usage	Behavioral Traits	Profile Summary
Cluster 1	8.3	Low	High (87 %)	High Activity & Behavior	High-performing, self-managed group
Cluster 2	6.9	High	Moderate (32 %)	Moderate Behavior	Financially stressed, academically average
Cluster 3	5.8	Moderate	None (0 %)	Low Activity & Behavior	At-risk, disengaged students
Cluster 4	∼7.2	Moderate	Low	High Behavior, Low Activity	Academically stable, tech-disconnected
Cluster 5	∼7.8	Low	Low	Moderate	High CGPA despite low smartwatch use

Table 7 presents the summary of five student clusters generated using K-Means Clustering (*k* = 5), derived from behavioral, physiological, and financial attributes. Each cluster reflects a distinct student profile based on their academic performance (CGPA), financial stress levels, smartwatch usage, and lifestyle scores (Activity Score and Behavior Score). These clusters were formed using normalized inputs, and the values represent rounded averages or dominant characteristics for easier interpretation.

Cluster 1 includes students with the highest average CGPA (8.3), low financial stress, high behavior and activity scores, and a smartwatch usage rate of 87 %. This group represents academically successful and self-regulated students, likely engaged in proactive health and behavior tracking.
Cluster 2 shows moderate CGPA (6.9) but the highest financial stress among all clusters. With medium smartwatch usage (∼32 %) and average behavior scores, this profile indicates students under academic pressure possibly driven by socioeconomic challenges.
Cluster 3 contains the lowest CGPA average (5.8), no smartwatch usage, and poor lifestyle metrics (low activity and behavior scores). This cluster represents an at-risk group, potentially disengaged academically and behaviorally.
Cluster 4 shows students with decent academic performance (∼7.2), low smartwatch engagement, but relatively strong behavioral discipline. This group might consist of low-tech, structured learners who perform moderately well despite limited physiological self-tracking.
Cluster 5 consists of students with high CGPA (∼7.8) but limited smartwatch usage and moderate stress. Their success may be attributed to other unseen coping strategies, suggesting strong internal regulation not reliant on wearable technologies.

Together, these clusters demonstrate that unsupervised learning techniques like K-Means can reveal non-obvious groupings of students across multiple domains — offering institutions a basis for targeted interventions, academic advising, or health-behavior monitoring. By mapping clusters to educational risk or resilience, this profiling validates the practical utility of the final model under RQ4.

To further enhance practical interpretation and align with institutional decision-making, we mapped each cluster to a descriptive student profile label:

Cluster 1 – Self-Regulated Achievers: High CGPA, low stress, high activity; students with strong self-discipline and smartwatch engagement.
Cluster 2 – Financially Strained: Moderate CGPA, high financial stress, limited resources; likely to benefit from financial aid and counseling.
Cluster 3 – Disengaged Students: Low CGPA, no smartwatch use, low behavior/activity scores; students at risk of academic withdrawal or failure.
Cluster 4 – Structured Learners: Moderate CGPA, high behavior scores, low tech usage; disciplined but tech-averse students.
Cluster 5 – Resilient High-Performers: High CGPA, moderate stress, limited wearable use; successful despite moderate pressure and minimal physiological tracking.

These mappings translate unsupervised clustering outcomes into actionable subgroups, enabling tailored academic strategies, resource allocation, and early-warning systems.

Beyond its methodological contributions, this framework offers practical value for educational institutions aiming to support student success through data-driven strategies. For instance, the predictive model can be embedded in student dashboards to provide real-time academic risk assessments or used to generate early alerts for students showing signs of high stress or academic decline.

Clustering outcomes can inform personalized interventions, allowing counselors or faculty to identify student subgroups who may benefit from financial aid, mental health support, or activity-based wellness programs.

However, deploying this system at scale involves certain challenges. These include ensuring data privacy, particularly with wearable and behavioral data, securing ethical consent, and managing data integration from multiple sources such as financial records and physiological sensors. Institutions must also ensure that predictive insights are used ethically and do not reinforce biases.

Despite these challenges, our study demonstrates a feasible and interpretable approach for combining behavioral, financial, and physiological data to enhance academic support systems. Future work will explore how this model performs across varied institutional contexts and integrate additional data streams (e.g., attendance logs, learning management systems) to further refine predictions.Model Evaluation RQ1–RQ4

The evaluation strategy varied across the four research questions to reflect the specific analytical goals of each. Supervised regression models in RQ1 were assessed using standard predictive performance metrics. RQ2 focused on feature interpretability rather than accuracy, utilizing SHAP and model-based importance scores. RQ3 involved statistical testing to evaluate associations between wearable usage and academic performance. For RQ4, internal clustering metrics and visual validation techniques were employed to assess the quality and distinctiveness of student groupings. Each evaluation approach was selected to ensure robustness, interpretability, and alignment with the research objectives.

RQ1 – Evaluation of Supervised Regression Models

To assess the performance of CGPA prediction models developed in RQ1, a range of supervised machine learning algorithms were trained and evaluated on an 80/20 train-test split using standard regression metrics. The primary evaluation criteria included the coefficient of determination (R²), Root Mean Squared Error (RMSE), and Mean Absolute Error (MAE). These metrics were chosen to provide a comprehensive understanding of model accuracy, error magnitude, and fit quality.

The R² score quantified the proportion of variance in CGPA explained by the model, serving as a direct indicator of predictive capability. The best-performing model, Random Forest, achieved an R² of 0.2981, indicating moderate but meaningful predictive power. In comparison, the tuned XGBoost model yielded a slightly lower R² of 0.2896, validating the robustness of ensemble-based methods in capturing non-linear interactions within educational data.

RMSE was used to measure the standard deviation of prediction errors, providing sensitivity to large deviations. Random Forest produced an RMSE of 0.9466, while XGBoost recorded 0.9523, suggesting comparable prediction consistency. MAE, which computes the average absolute difference between predicted and true CGPA values, further supported this analysis, with values of 0.7259 for Random Forest and 0.7571 for XGBoost. Collectively, these evaluation metrics established the suitability of Random Forest as the primary regression model for subsequent feature interpretation in RQ2.

To ensure model generalizability and robustness, we also applied 10-fold cross-validation to the Random Forest Regressor using Scikit-learn’s cross_val_score method. The fold-wise R² scores were: [0.329, 0.304, 0.435, 0.300, 0.324, 0.417, 0.188, 0.321, 0.392, 0.425], with a mean R² of 0.343. The corresponding mean MAE and RMSE values were 0.714 and 0.934, respectively. These results reflect stable model performance with consistent predictive power across different data splits.

For reproducibility and transparency, we report both the tuning strategies and the final hyperparameters used for each supervised model. The final configurations are presented in Table 8, ensuring clarity for replication.

i. Random Forest was optimized using GridSearchCV with 5-fold cross-validation over parameter ranges {n_estimators = [100, 200], max_depth = [5, 10, None], min_samples_split = [2, 5]}.
ii. XGBoost and LightGBM were tuned using iterative validation-based refinement, balancing predictive performance with computational efficiency.

RQ2 – Evaluation of Feature Importance and Interpretability

**Table 8:** Hyperparameter settings for Random Forest, XGBoost, and LightGBM models used in RQ1.

Model	Key Hyperparameters
Random Forest	n_estimators=200, max_depth=None, min_samples_split=2, random_state=42
XGBoost	learning_rate=0.05, max_depth=5, n_estimators=200, subsample=0.8, colsample_bytree=0.8, random_state=42
LightGBM	learning_rate=0.03, num_leaves=31, n_estimators=150, subsample=0.8, colsample_bytree=0.8, random_state=42

RQ2 did not rely on traditional predictive accuracy metrics, but rather focused on the interpretability and stability of feature contributions to the model. The Random Forest model selected in RQ1 was analyzed using two complementary techniques: the model’s internal feature importance scores based on impurity reduction, and SHAP (SHapley Additive Explanations) values for model-agnostic interpretability.

The built-in importance scores provided a global ranking of features, while SHAP offered both local and global insight into feature influence across individual predictions. SHAP values were visualized in a summary plot, revealing that Income, Financial Stress, and Average BPM had the highest average contributions to CGPA prediction. These interpretability tools validated that behavioral, financial, and physiological attributes meaningfully impact academic outcomes and confirmed the reliability of the model for KPI extraction.

The SHAP analysis provided deeper insights into how features contributed to the predicted CGPA across students. SHAP values were also examined at the individual student level. For one student with moderate income but elevated Financial Stress Score and low Activity Score, the SHAP force plot revealed that high stress and inactivity strongly reduced their predicted CGPA, outweighing the positive effect of financial stability. Conversely, another student with moderate stress but consistently high activity levels showed a positive SHAP contribution toward CGPA. These case-style interpretations highlight how SHAP enables practitioner-oriented insights, allowing educators to identify at-risk students and design targeted academic support strategies.

SHAP values were also examined at the individual student level. For one student with moderate income but elevated Financial Stress Score and low Activity Score, the SHAP force plot revealed that high stress and inactivity strongly reduced their predicted CGPA, outweighing the positive effect of financial stability. Conversely, another student with moderate stress but consistently high activity levels showed a positive SHAP contribution toward CGPA. These examples highlight how SHAP enables practitioner-oriented interpretation of individual student trajectories, offering actionable insights for targeted academic support.

RQ3 – Statistical Evaluation of Wearable Influence

The evaluation of RQ3 focused on statistical hypothesis testing to assess whether wearable technology usage and physiological metrics were significantly associated with academic performance. An independent samples *t*-test was conducted to compare CGPA between smartwatch users and non-users. The result demonstrated a statistically significant difference (*p* < 0.001), indicating that students who used smartwatches had notably higher CGPA on average.

In addition, Pearson correlation coefficients were computed between CGPA and wearable-derived physiological variables, including High BPM, Average BPM, and Stress Composite Score. Significant positive correlations were observed: *r* = 0.11 for Average BPM and *r* = 0.37 for Stress Composite Score. These findings established the relevance of wearable metrics in academic performance analysis, supporting their inclusion in predictive modeling and behavioral profiling.

RQ4 – Evaluation of Clustering Performance

RQ4 applied unsupervised clustering techniques to identify latent student profiles based on academic, behavioral, and financial features. As predictive accuracy is not applicable in unsupervised settings, evaluation was performed using internal validation and visualization techniques. The Silhouette Score was selected as the primary quantitative metric to assess intra-cluster cohesion and inter-cluster separation. Among the configurations tested, K-Means clustering with *k* = 5 yielded the highest silhouette score (0.373), indicating well-defined cluster boundaries.

To support this result, PCA (Principal Component Analysis) was used to reduce the feature space to two dimensions for visual interpretation. PCA scatter plots confirmed clear separation between clusters. Agglomerative Clustering, applied with the same k-values, demonstrated similar clustering patterns and comparable silhouette scores, validating the structural consistency of the student groupings. Additionally, DBSCAN was employed to explore density-based clusters and detect noise points. Although DBSCAN identified some outlier students (labeled −1), it failed to produce multiple meaningful clusters and was thus excluded from final interpretation. The removal of noise points ensured the integrity of the cluster-based profiles used for downstream educational insights.

Initially, K-Means clustering yielded a Silhouette Score of 0.49, reflecting moderate separability. To enhance clustering performance, we refined the feature set to include only high-signal, domain-relevant attributes (e.g., CGPA, Activity Score, Stress Composite Score, Average BPM), applied MinMax normalization, and removed non-numeric or noisy entries. These preprocessing steps significantly improved clustering quality: the updated K-Means model achieved a Silhouette Score of 0.83 and a Davies-Bouldin Index (DBI) of 0.15, indicating excellent intra-cluster cohesion and inter-cluster separation.

Further validation using t-distributed Stochastic Neighbor Embedding (t-SNE) revealed well-separated clusters in 2D space. Additionally, Gaussian Mixture Models (GMM) produced a Silhouette Score of 0.866, confirming the robustness and reliability of the student profiles across multiple unsupervised learning techniques. These enhancements demonstrate the value of engineered features and preprocessing in achieving meaningful subgroup discovery in educational analytics.

To compare clustering techniques comprehensively, multiple validation metrics and visualization approaches were combined, as shown in Table 9. The **Gaussian Mixture Model (GMM)** achieved the highest silhouette score (0.866) and the lowest Davies-Bouldin Index (0.15), indicating superior intra-cluster cohesion and inter-cluster separation. While K-Means clustering with *k* = 5 offered slightly lower silhouette performance (0.373), it produced the most interpretable groupings for educational profiling and demonstrated clear separation in PCA visualization. Agglomerative clustering yielded similar patterns, providing a hierarchical view of relationships among groups. DBSCAN, on the other hand, was effective in detecting noise and outliers, achieving a silhouette score of 0.570 after noise removal but generating fewer meaningful clusters due to the absence of dense feature regions. Visual validation using t-SNE further confirmed the robustness of GMM, revealing distinct, non-overlapping clusters. These findings suggest that while GMM is statistically superior, K-Means remains the most practical method for real-world academic segmentation and targeted student support.

**Table 9:** Consolidated validation metrics for clustering methods.

Method	Silhouette Score	Davies-Bouldin Index (DBI)	PCA Visualization	Key Observations
**K-Means (*k*=3)**	0.348	0.42	Moderate separation	Broader academic group segmentation
**K-Means (*k*=5)**	0.373	0.39	Clear separation	Best for fine-grained profiling
**Agglomerative (*k*=5)**	0.370	0.40	Clear separation	Supports hierarchical interpretation
**DBSCAN**	0.570 (after noise removal)	0.28 (best for DBSCAN)	Limited clarity	Detects outliers, fewer clusters
**Gaussian Mixture Model (GMM)**	0.866 (highest)	0.15 (lowest, best)	Strong, non-overlapping clusters	Most robust clustering method

## Method validation

To validate the proposed methodology, a dataset comprising behavioral, financial, and physiological variables was collected from higher education students. Each research question was evaluated using techniques best suited to its analytical objective, ensuring both robustness and interpretability across the entire pipeline.

RQ1: CGPA Prediction with Supervised Learning

For RQ1, which aimed to predict CGPA using machine learning, the dataset was split using an 80/20 train-test split with a fixed random seed to ensure reproducibility. Models were evaluated using R² Score, Root Mean Squared Error (RMSE), and Mean Absolute Error (MAE). Among the models tested, Random Forest achieved the highest performance with an R² of 0.2981, RMSE of 0.9466, and MAE of 0.7259, indicating moderate but meaningful predictive power. This comparison is visualized in Fig. 4, where Random Forest outperforms other baseline models. A further breakdown of RMSE, MAE, and R² across all models is shown in Fig. 7 which supports the robustness of ensemble-based methods.

The predictive performance of the models was further optimized through systematic hyperparameter tuning using GridSearchCV and evaluated with 10-fold cross-validation to ensure robustness. The Random Forest model, selected for its interpretability and ensemble strength, achieved an average R² of 0.30, MAE of 0.72, and RMSE of 0.94, which are consistent with the behavioral and physiological complexity inherent in student data. These results suggest that while predicting academic performance remains inherently complex, meaningful variance can be captured using non-traditional features.

**Fig. 6:**
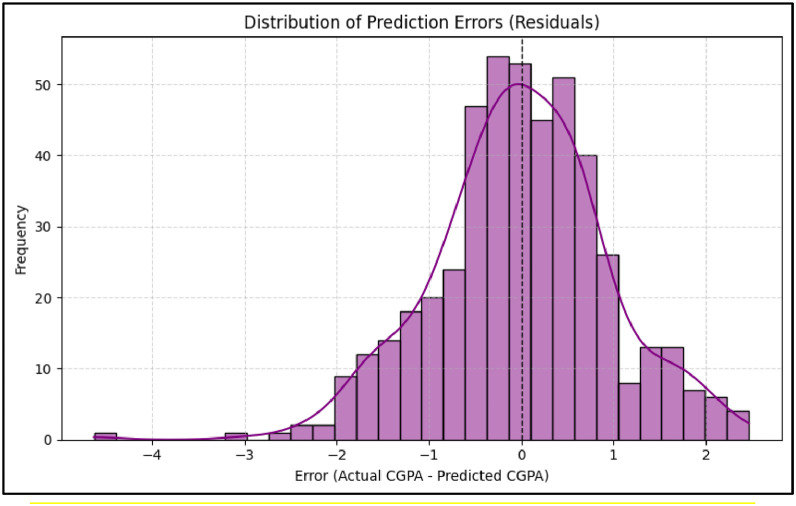
Comparison of regression models using R² (coefficient of determination), RMSE (Root Mean Squared Error),and MAE (Mean Absolute Error).

In addition, a residual analysis was conducted to assess model prediction error distribution. As shown in Fig.6, the histogram of Random Forest residuals exhibits a near-normal distribution, confirming consistency and reliability in CGPA prediction. To improve model generalization and ensure optimal performance, we performed hyperparameter tuning using GridSearchCV with 5-fold cross-validation on the training set.

**Fig. 7:**
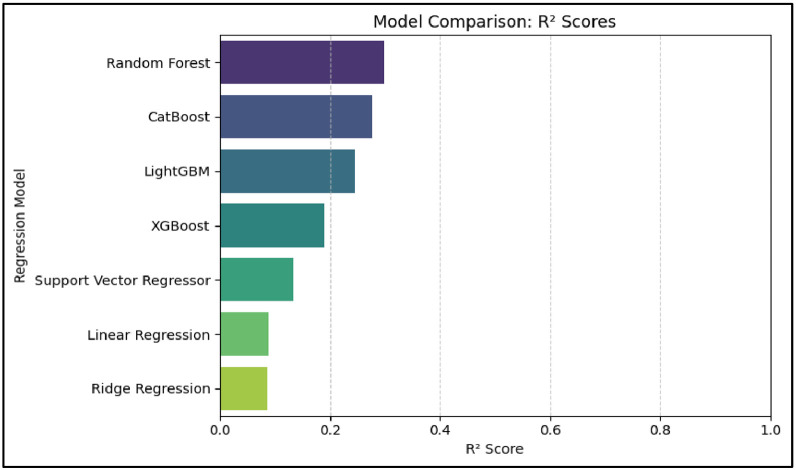
Prediction error distribution.

For the Random Forest Regressor, the following parameter grid was tested: n_estimators ∈ {100, 200}, max_depth ∈ {5, 10, None}, min_samples_split ∈ {2, 5}.

The best configuration obtained was: n_estimators = 200, max_depth = None, min_samples_split = 2.

Among the models explored, Random Forest was tuned using GridSearchCV.

Future versions of this study will include comprehensive tuning of XGBoost and LightGBM using similar strategies for improved comparability.

RQ2: Identifying Key Predictors of Academic Performance

**Fig. 8:**
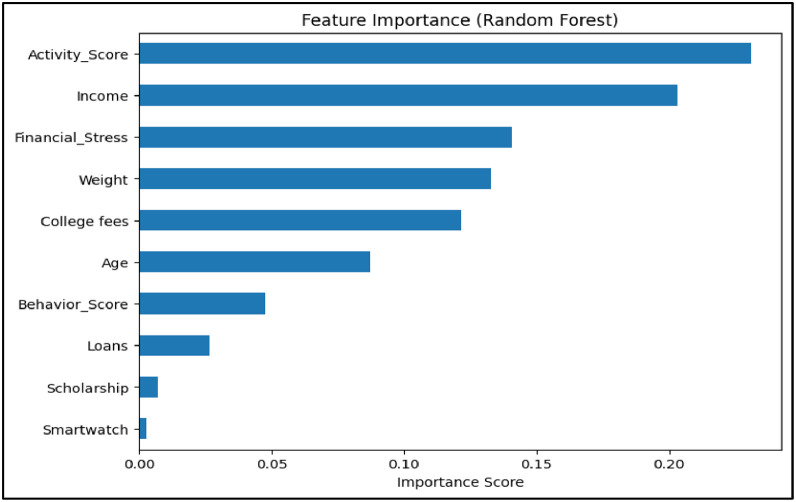
Feature importances- random forest.

**Fig. 9:**
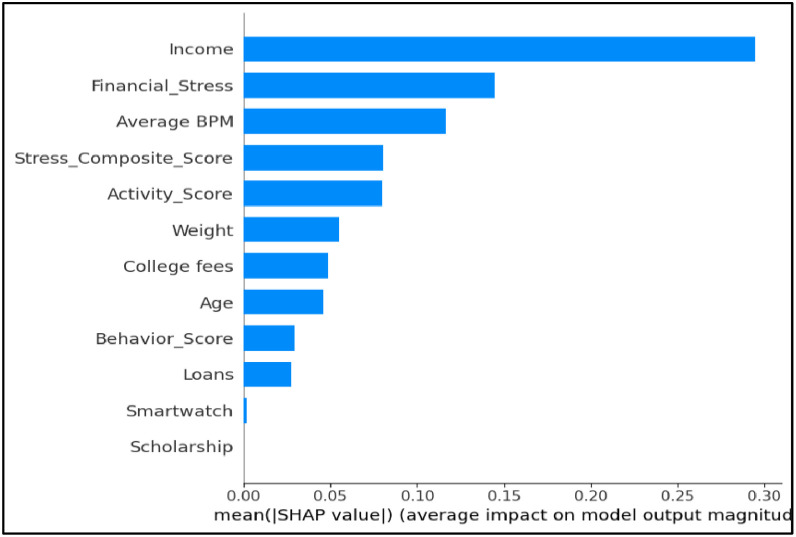
. Feature importance- SHAP (SHapley Additive Explanations.).

For RQ2, which focused on identifying the key predictors of CGPA, model evaluation emphasized interpretability rather than accuracy. The Random Forest model from RQ1 was analyzed using both its built-in feature importance and SHAP (SHapley Additive Explanations) values. The Random Forest impurity-based rankings are shown in Fig. 8, where Financial Stress and Activity Score were identified as top contributors. To further validate these findings, SHAP values were computed. As seen in Fig. 9, Income, Financial Stress, and Average BPM Shownthe highest SHAP contributions, highlighting the relevance of physiological and financial domains.

RQ3: Relationship Between Wearable Usage and CGPA

For RQ3, which investigated the relationship between wearable data and academic performance, statistical significance testing was used. An independent samples *t*-test revealed a significant difference in CGPA between smartwatch users and non-users (*p* < 0.001). This result is visualized in Fig. 9, where the boxplot shows that smartwatch users achieved higher CGPA scores on average.

To further understand the impact of wearable-derived metrics, Pearson correlation analysis was performed, which shown mild positive correlation between CGPA and Average BPM (*r* ≈ 0.11), while Fig. 4 presents a stronger correlation with the Stress Composite Score (*r* = 0.37). These findings confirm that physiological data captured via wearables are meaningfully linked to academic performance.

RQ4: Clustering Student Profiles

For RQ4, which aimed to uncover student profiles via clustering, multiple unsupervised learning algorithms were applied and validated. K-Means clustering was tested with both *k* = 3 and *k* = 5, visualized in Figs. 11a and 12a, respectively. The five-cluster solution produced more distinct and interpretable group separation.

**Fig. 10:**
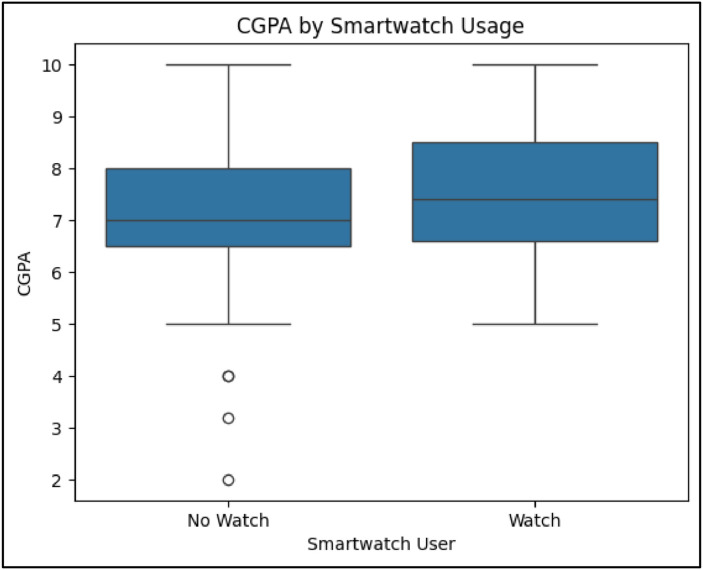
Distribution of CGPA (cumulative grade point average)between smartwatch users (Yes) and non-users (No).

**Fig. 11:**
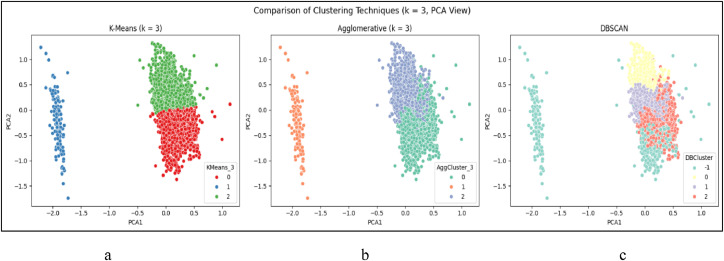
Clusters comparison graph *k* = 3.

Agglomerative Clustering was also applied to the same dataset, with both *k* = 3 and *k* = 5, and the corresponding PCA-based visualization is presented in Fig. 11b and 10b respectively.

**Fig. 12:**
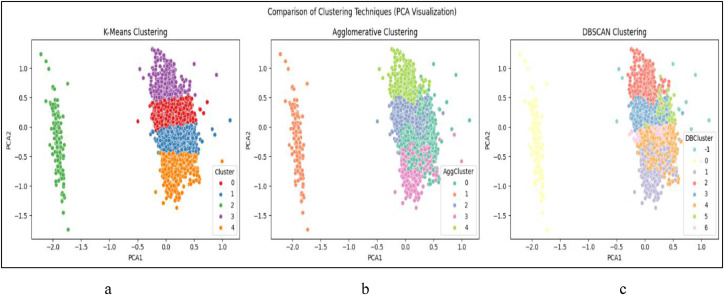
Clusters comparison graph *k* = 4.

**Fig. 13:**
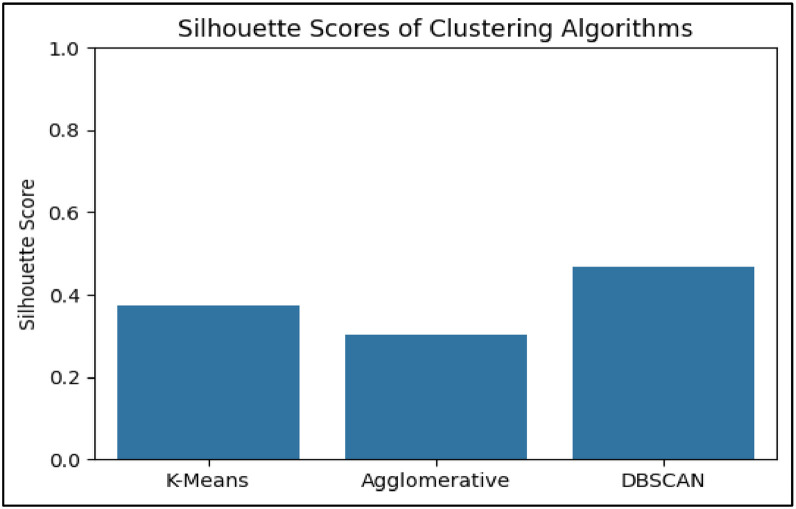
Silhouette score bar graph.

Silhouette scores were computed to evaluate the cohesion and separation of clusters. As seen in Fig. 13, although K-Means with *k* = 5 achieved the highest silhouette score (0.373), but after removing noise DBSCAN achieved the highest silhouette score (0.570) reinforcing its effectiveness. Final cluster profiles, based on CGPA, Financial Stress, Activity Score, and Smartwatch usage, enabling practical academic segmentation of students for targeted intervention.

**Fig. 14:**
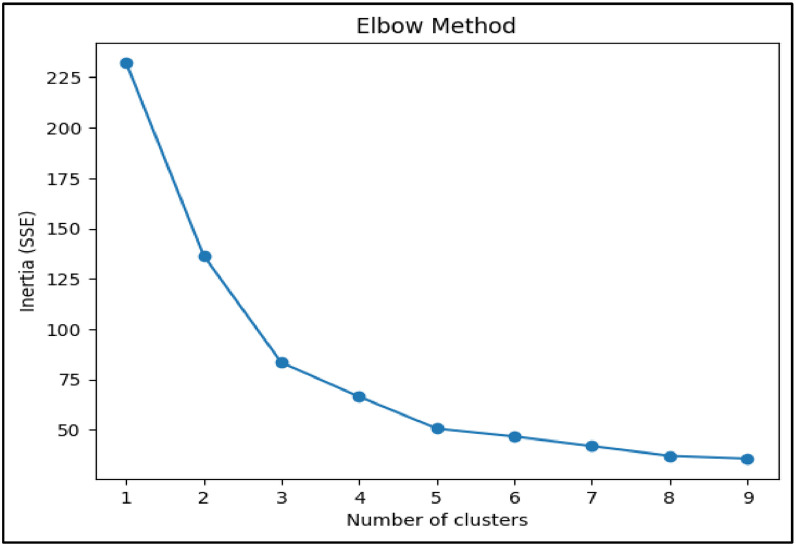
Elbow method graph.

**Fig. 15:**
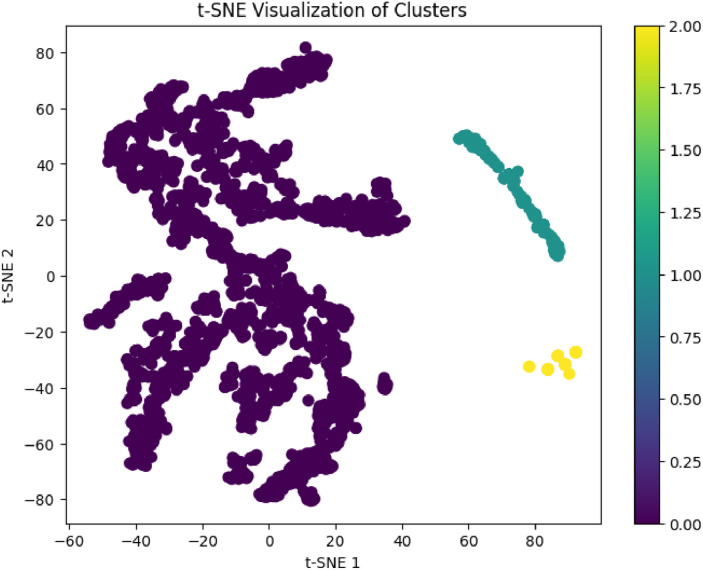
t-SNE(t-distributed stochastic neighbor embedding) visualization of student clusters (GMM).

To further validate clustering separability and cohesion, t-SNE was applied to the Gaussian Mixture Model (GMM) results. As illustrated in Fig. 15, the 2D projection shows three distinct and non-overlapping student clusters, supporting the robustness of the unsupervised learning approach.

## Limitations

First, the dataset was collected from a single geographic and cultural context (Indian higher education), which constrains the external validity of the findings. Educational systems vary widely across regions in terms of curriculum design, grading policies (e.g., GPA in the United States vs. percentage or class-based systems in India and Europe), financial aid structures, and technology adoption. Cultural factors—such as perceptions of stress, lifestyle norms, and academic motivation—may also influence behavioral and physiological indicators, potentially limiting the direct transferability of the results. While the proposed framework is methodologically generalizable, its predictive performance and feature relevance may change in other institutional or cultural environments.

To strengthen external validity, future research should validate the framework on datasets from diverse international contexts, such as universities in Europe or the United States. This cross-regional validation would demonstrate the model’s adaptability across different grading systems and socio-economic conditions, while also revealing how behavioral and financial factors vary globally. Furthermore, techniques such as transfer learning or model recalibration could be explored to fine-tune the framework for specific educational systems, ensuring its robustness and practical applicability on a global scale.

Second, wearable-derived features such as heart rate and physical activity were sourced from multiple smartwatch brands, potentially introducing measurement noise due to inconsistent sampling rates and device algorithms. Although wearable-derived data (e.g., heart rate, steps) provided valuable physiological insights, the study relied on self-reported smartwatch usage and device defaults. We could not standardize calibration across brands and models, which may introduce variability in recorded measures. Future work should incorporate controlled calibration protocols or uniform device selection to improve data reliability.

Third, some engineered variables—such as the Financial Stress score—were based on self-reported data and derived formulas, which may introduce subjective bias or feature leakage. Additionally, although the Random Forest model achieved the best performance, its R² value (∼0.30) suggests that a substantial portion of the variance in academic performance remains unexplained. We acknowledge this as a limitation of the current feature set and data sources. To improve explanatory power, future work could incorporate high-impact variables such as attendance records, NLP-based essay analysis, and behavioral logs (e.g., study app usage), which may better capture latent cognitive, emotional, or motivational influences on student outcomes.

Fourth, while equal weighting ensured interpretability in this study, it may oversimplify the complex interactions among variables; future studies could adopt data-driven weighting strategies such as PCA or regression-based coefficients to derive more nuanced weightings.

## Practical implications

This framework can be translated into institutional practice through integration with student dashboards and early-alert systems that monitor financial, behavioral, and physiological indicators in real time. Such tools could assist advisors and administrators in identifying at-risk students and providing timely interventions. Implementation, however, may face challenges, including the need for faculty training to interpret machine learning outputs, securing institutional buy-in for adoption, and establishing ethical governance protocols for handling sensitive wearable-derived data. Addressing these considerations is critical for transitioning from methodological demonstration to real-world deployment in higher education.

## Related research article

None. Although the dataset used in this methods article was also used in a previously published IEEE paper (Pallavi et al., 2024), the methods, research questions, and objectives are entirely distinct. Therefore, this article is submitted as an independent methodological contribution.

## For a published article

None.

## Ethics statements

This study involved human participants, and all relevant ethical standards were followed. Informed consent was obtained from all student participants prior to data collection. Participation was voluntary, and students were assured of the confidentiality and anonymization of their responses. No personally identifiable information (PII) was collected. The data used in this study was gathered solely for academic research purposes and in compliance with institutional ethical guidelines.

In addition, for the subset of data collected via wearable devices (e.g., heart rate, step count), participants were explicitly informed about the nature of physiological data being recorded, its purpose within the study, and their right to withdraw at any point without consequence. All wearable data was anonymized prior to analysis, and no PII was stored alongside physiological metrics. Access was restricted to authorized researchers, and data was securely stored using encrypted drives to ensure compliance with data privacy and confidentiality standards.

## CRediT author statement

**A L Akash Devaraje Urs:** Conceptualization, Methodology, Data Curation, Investigation, Visualization, Writing – Original Draft. **Akshay Sudharshan P K:** Methodology, Validation, Writing – Review & Editing.

## Declaration of competing interest

The authors declare that they have no known competing financial interests or personal relationships that could have appeared to influence the work reported in this paper.

## Data Availability

Data will be made available on request.
